# An Analysis of Algebraic Codes over Lattice Valued Intuitionistic Fuzzy Type-3 *R*-Submodules

**DOI:** 10.1155/2022/8148284

**Published:** 2022-06-23

**Authors:** Asra Riaz, Sajida Kousar, Nasreen Kausar, Dragan Pamucar, Gezahagne Mulat Addis

**Affiliations:** ^1^Department of Mathematics and Statistics, International Islamic University, Islamabad, Pakistan; ^2^Department of Mathematics, Faculty of Arts and Sciences, Yildiz Technical University, Esenler 34210, Istanbul, Turkey; ^3^Department of Logistics, University of Defence, Belgrade, Serbia; ^4^Department of Mathematics, University of Gondar, P.O. Box 196, Gondar, Ethiopia

## Abstract

In the last few decades, the algebraic coding theory found widespread applications in various disciplines due to its rich fascinating mathematical structure. Linear codes, the basic codes in coding theory, are significant in data transmission. In this article, the authors' aim is to enlighten the reader about the role of linear codes in a fuzzy environment. Thus, the reader will be aware of linear codes over lattice valued intuitionistic fuzzy type-3 (LIF-3) *R*-submodule and *α*-intuitionistic fuzzy (*α*-IF) submodule. The proof that the level set of LIF-3 is contained in the level set of *α*-IF is given, and it is exclusively employed to define linear codes over *α*-IF submodule. Further, *α*-IF cyclic codes are presented along with their fundamental properties. Finally, an application based on genetic code is presented, and it is found that the technique of defining codes over *α*-IF submodule is entirely applicable in this scenario. More specifically, a mapping from the *ℤ*_64_ module to a lattice *L* (comprising 64 codons) is considered, and *α*-IF codes are defined along with the respective degrees.

## 1. Introduction

The process of data hauling in the recent decades has been significantly escalated. To widen the utility of communication networks, the employment of intricate technologies such as wavelength division multiplexing has been enforced through the development in signal processing. Due to these advancements, a dire obstacle of unreliability in data transmission is faced; this may be impacted by the means of transmission or any other reason. For the maximum efficacy, there is an urge to control and process the glitches with the assurance of data transmission. To circumvent this problem, one may utilize the understanding of the rules to digitally interpret and store information. The system of rules for digital data transmission is known as a code. The algorithm that represents a sequence of numbers to detect and correct an error is called an error-correcting code. Initially, codes are defined over finite field *𝔽*_2_, among which the binary codes are the simplest. Say, in a binary field *𝔽*_2_, two codewords 110 and 111 are considered; then, both codewords comprise three bits. Now, when a message carrying 111 is sent in binary coding, the received message should contain 111 theoretically. Often, the transmission is corrupted, and the received message could be carrying 110. This error can be corrected by understanding the coding theory, and data transmission can be made more reliable and accurate. Linear codes are the most basic error-correcting codes which are the subspaces of a vector space. These codes are crucial to data transmission and data storage [1]. Just like vector spaces, codes are defined over groups and applied in channel and source coding [2–4].

Now, when dealing with the data transmission, one cannot always get crisp zeros and ones with data transmission ambiguity being an often occurring scenario. There may be plenty of reasons behind the emergence of uncertainty. These may be lack of knowledge, chance, imprecision, lack of information, and complexity. The imprecision inherent in the process can be dealt with by using fuzzy logic. Fuzzy sets (IF) are opposite to ordinary sets (crisp sets). In crisp set theory, things are categorized by the values 0 and 1, but the full membership and nonmembership are determined in a fuzzy set. This technique copes mathematically with the vagueness of determining boundaries by assigning grades of membership to the elements. Fuzzy set theory as a generalization of the classical set theory was established by Zadeh [5] in which each element under consideration is graded with a membership value ranging between zero and one. For instance, a membership extent *μ*_*A*_(*x*)=0.7 shows that *x* belongs to set *A* with the degree 0.7 on a scale whereas zero indicates that there is no membership and one suggests complete membership. He also defined various properties of a fuzzy set such as union, intersection, and complements. This theory of fuzzy set was proved to be more effective against ambiguity. Atanassov [6] proposed the notion of an intuitionistic fuzzy set (IFS) which is a generalized concept of the fuzzy set governed by incorporating the degree of nonmembership along with the degree of membership. Goguen [7] discussed order structure and L-fuzzy set which is the generalization of fuzzy subset *X*, as a function from subset *X* to a lattice *L*. Atanassov and Stoeva [8] proposed the definition of lattice valued intuitionistic fuzzy set (LIFS-1) where a complete lattice with a unary operation *N* : *L*⟶*L* is considered. Gerstenkorn and Tepa $\overset{⌣}{v}$ cev $\dot{i}$ [9] extended the concept formulated by Atanassov and defined LIFS-2 by replacing unary operation with unary operator *N* by a linearization function *ℓ* : *L*⟶[0,1]. The choice of linearization creates a problem; to overcome this drawback, it is replaced by Lattice homomorphism *α* : *L*⟶[0,1], and it is said to be LIFS-3.

The Fuzzy set is the most suitable framework to model uncertain data which plays an important role in the data transmission. The vagueness in data transmission can be handled by involving fuzzy theoretic concepts in coding structures. Many researchers have worked on and proposed significant results related to this field. Kaenel and Pierre [10] considered *n*-dimensional vector space and defined fuzzy codes which are fuzzy subsets of *n*-tuples over the field. Hamming distance is also defined between two fuzzy codewords. Hall and Dial [11] investigated whether the distance between the fuzzy codeword and fuzzy subsets of *n*-tuples depends on the dimension of the space and distance between codewords which are non-fuzzy.

$\overset{ˇ}{S}$ *e* $\overset{ˇ}{s}$ elja and Tepav $\overset{ˇ}{c}$ evi $\overset{´}{c}$ [12] introduced another method of involving fuzzy theory in coding based on defining a map $\bar{A}$ from a non-empty set *S*={1,2,…, *n*} to partially ordered set *P*. $\overset{⌣}{S}$*e*$\overset{⌣}{s}$ elja et al. [13] used the concept and defined binary block codes over lattice valued fuzzy sets (*L*-fuzzy sets). $\overset{⌣}{Z}$*i*$\overset{⌣}{z}$ ovi $\dot{c}$ and Lazarevi $\dot{c}$ [14] discussed the length and cardinality of block codes over *L*-fuzzy sets. Amudhambigai and Neeraja [15] examined fuzzy codes and defined some basic operations including fuzzy complement, fuzzy intersection, and fuzzy union of fuzzy codes. Tsafack et al. [16] considered the Galois ring. They presented fuzzy linear codes and fuzzy cyclic codes. Shijina [17] investigated the notion of multi-fuzzy code, which is defined as multi-fuzzy subset of *n*-tuples over *F*, and proposed fundamental properties of these codes. Hamming distance of multi-fuzzy codes was also presented.

The algebraic codes are also studied in various other disciplines and have a wide range of applications in numerous fields like data compression, cryptography, network processing, and neuroscience. Considerable work relevant to these fields has been done. Timm and Lapish [18] studied the encoding of information in neuroscience. To understand brain functions, it is important to know how a neural system integrates, encodes, and computes information. Various models were also analyzed to illustrate the strengths of the information theory analysis. Dong and Li [19] considered the linear network coding based qualitative communication and proved its importance. Kong et al. [20] discussed Alamouti code based on block repetition in FBMC/OQAM systems. A novel block-wise Alamouti code, where a repeated block is designed to remove the imaginary interference among FBMC/OQAM symbols, was presented. Marani [21] examined *G*-invariant codes from primitive permutation representations of Mathieu groups M24 and M23.

In this article, LIFS-3 codes over *R*-submodule are defined. Module is a useful algebraic structure introduced as an extensions of vector space where scalars are from the arbitrary ring instead of a field. Fuzzy logic has significant importance in the theory of modules and rings. Remarkable work has been done by the researchers in this field. Further, Negoita and Ralescu [22] presented the concept of fuzzy modules. Many other researchers have also worked in this field and studied fuzzy modules. Zahedi [23] studied *L*-fuzzy modules. He also presented basic operations on L-fuzzy modules such as addition and intersection and proved some properties related to these operations. Sharma [24] introduced the concept of an intuitionistic fuzzy module over an intuitionistic fuzzy ring. Sharma and Kanchan [25] in the continuation of this research work on the intuitionistic *L*-fuzzy modules discussed some important results related to the L-fuzzy module and L-prime fuzzy module.

This article is based on the construction of linear codes over lattice valued intuitionistic fuzzy type-3 *R*-submodule. Already existing fuzzy linear codes are defined over *R*-submodule which involves only the degree of membership. Although fuzzy set theory provides a convenient way to model uncertain data, in some situations, these are not more helpful when we need some extra information along with a membership degree. Lattice valued intuitionistic fuzzy sets which are the extension of fuzzy sets provide an effective tool to study the case of vagueness and have a significant role in various branches of mathematics such as group theory and module theory. Linear codes defined over lattice valued intuitionistic fuzzy type-3 *R*-submodule are more efficient as compared to ordinary fuzzy linear code over submodule because these involve both the degrees of belongingness and non-belongingness.

## 2. Preliminaries

In this section, some basic definitions will be discussed.

### 2.1. Linear Codes

Let *𝔽* be a finite field; then, *𝔽*^*n*^ is an *n*-dimensional vector space over *𝔽*. A code *C* over *𝔽* is simply a subset of *𝔽*^*n*^. The elements of code *C* are called the codewords, and these codewords are represented as (*a*_1_, *a*_2_,…, *a*_*n*_). A code *C* is said to be linear code over field *𝔽* if *u*, *v* ∈ *C*; then, *αu*+*βv* ∈ *C* for all *α*, *β* ∈ *C*. Thus, *C* is a subspace of *𝔽*^*n*^. If *𝔽*=*ℤ*_2_={0,1} under addition and multiplication modulo 2, then the code is said to be a binary code, and if the code is linear, then it is called a binary linear code. Let *𝔽* be a finite field. A code *C* is called cyclic if the cyclic shift of each codeword in *C* is also a member in *C*. Let *C* be a code over *𝔽*. Then, the corresponding dual code is defined to be

(1) $$\begin{array}{c} C^{⊥}=\left\{u\in F:u.v=0,\forall v\in C\right\}. \end{array}$$

Thus, the dual code consists of all the codewords that are orthogonal to every codeword in *C*.

### 2.2. Lattice Order Set

A relation ϱ on a non-empty set is *S* is a subset of *S* × *S*. If (*x*, *y*) belongs to a relation ϱ, this implies *x* is related to *y*, or we can write it as *x*ϱ*y*. A relation on a set *S* is said to be a partial order relation if it satisfies three properties, that is, reflexive property (*x* ≤ *x*,  for all *x* ∈ *X*), antisymmetric property (*x* ≤ *y*∧*y* ≤ *x* imply *x*=*y*), and transitive property (*x* ≤ *y*∧*y* ≤ *z* imply *x* ≤ *z*).

Consider two non-empty sets *A* and *B*, where *B* is a subset of *A*. An element *a* ∈ *A* is said to be the upper bound of *B* provided that

(2) $$\begin{array}{c} b\leq a\text{ for all }b\in B. \end{array}$$

In addition, the element *a* is said to be lower bound of set *B* if

(3) $$\begin{array}{c} b\geq a\text{ for all }b\in B. \end{array}$$

If *U* is the set of all upper bounds, then the least element of this set is called a supremum or join (∨) and if *L* is the set of all lower bounds, then the greatest element of set *L* is called an infimum or meet (∧).

A set together with partial order is said to be a lattice if every set of two elements have supremum and infimum.

### 2.3. Lattice Valued Intuitionistic Fuzzy Set

The notion of fuzzy set introduced by Zadeh [1] is an extension of an ordinary set. Given universe *S*, a fuzzy set *A* is an ordered set (element of universe, degree of that element). Mathematically,

(4) $$\begin{array}{c} A=\left\{\left(x,\mu \left(x\right)\right):x\in S\right\}. \end{array}$$

The grade of membership indicates the confirmation of an element that belongs to that set, but it does not give any information about the element which does not belong to that set. For this purpose, a generalization of the fuzzy set was introduced. For a given universe *S*, an intuitionistic fuzzy set is a triplet that consists of an element of universe, value of membership of that element, and value of nonmembership of that element. Mathematically, it can be represented as

(5) $$\begin{array}{c} A=\left\{\langle x,\mu \left(x\right),\nu \left(x\right)\rangle :x\in S\right\}\text{,} \end{array}$$

with 0 ≤ *μ*(*x*)+*ν*(*x*) ≤ 1.

Let *S* be a non-empty set and *L* be a lattice. Consider *f* : *S*⟶*L*, *g* : *S*⟶*L*, where *f* and *g* are membership and nonmembership functions; then, a lattice valued intuitionistic fuzzy set of type-1 (LIFS-1) is the set (*S*, *L*, *f*, *g*, *N*), where *N* is an involutive order reversing unary operator on *L* such that *f*(*x*) ≤ *N*(*g*(*x*)).

Let *S* be a non-empty set and *L* be a lattice. Consider *f* : *S*⟶*L*, *g* : *S*⟶*L*; then, a lattice valued intuitionistic fuzzy set of type-2 (LIFS-2) is the set (*S*, *L*, *f*, *g*, *ℓ*), where *ℓ* : *L*⟶[0,1] is a linearization function satisfying *ℓ*(*f*(*x*))+*ℓ*(*g*(*x*)) ≤ 1.

Let *S* be a non-empty set and *L* be a complete lattice with top element *T* and bottom element *B*. Consider *f* : *S*⟶*L*, *g* : *S*⟶*L* which are membership and nonmembership functions; then, a lattice valued intuitionistic fuzzy set of type-3 (LIFS-3) is the set (*S*, *L*, *f*, *g*, *α*), where *α* : *L*⟶[0,1] is a lattice homomorphism with *α*(*T*)=1, *α*(*B*)=0 satisfying

(6) $$\begin{array}{c} \alpha \left(\ell_{1}\wedge \ell_{2}\right)=\alpha \left(\ell_{1}\right)\wedge \alpha \left(\ell_{2}\right),\\ \alpha \left(\ell_{1}\vee \ell_{2}\right)=\alpha \left(\ell_{1}\right)\vee \alpha \left(\ell_{2}\right)\;\forall \ell_{1},\ell_{2}\in L\text{,} \end{array}$$

and *α*(*f*(*s*))+*α*(*g*(*s*)) ≤ 1 for all *s* ∈ *S*.

### 2.4. *R*-Module

Ring theory is one of the extensions of group theory that encompasses a broad set of present study topics in mathematics, computer science, and mathematical/theoretical physics. They have a wide range of applications in the studies of geometric objects and topology, and their connections to other fields of algebra are quite well understood in several contexts. A ring *R* is a set equipped with two binary operations, namely, addition + and multiplication · and satisfying the following axioms:

1. (*R*, +) is an Abelian group.
2. (*R*, ·)⟶ is a semigroup.
3. Multiplication is distributive over addition; that is, *a* · (*b*+*c*)=*a* · *b*+*a* · *c* and (*b*+*c*) · *a*=*b* · *a*+*c* · *a* for all *a*, *b*, *c* ∈ *R*.

The ring *R* is called commutative if *a* · *b*=*b* · *a* for all *a*, *b* ∈ *R*. Consider a commutative ring *R*. An *R*-module is a set *M* together with a binary operation addition ‘⊕' and scalar multiplication ‘*∗*', where *∗* : *R* × *M*⟶*M*; then, for all *r*, *s* ∈ *R*, *a*,  *b* ∈ *M*, we have the following:

1. *r*(*a* ⊕ *b*)=*ra* ⊕ *rb*.
2. (*r* ⊕ *s*)*a*=*ra* ⊕ *sa*.
3. (*rs*)*∗a*=*r*(*s∗a*).
4. 1_*R*_*a*=*a* where 1_*R*_ is the multiplication identity of the ring *R*.

Let *R*=*ℤ*_*p*^*k*^_ be a ring; then, *ℤ*_*p*^*k*^_^*n*^ is an *R*-module.

## 3. Lattice Valued Intuitionistic Fuzzy Type-3 *R*-Submodule

If *A*=(*M*, *f*, *g*, *L*, *α*) is a lattice valued intuitionistic fuzzy subset of type-3, where *f* : *M*⟶*L* and *g* : *M*⟶*L*, then *A* is called an LIF-3 submodule of *M* if for *r* ∈ *R* and *a*, *b* ∈ *M* we have the following:

1. *f*(*a*+*b*) ≥ *f*(*a*)∧*f*(*b*).
2. *f*(*r*.*a*) ≥ *f*(*a*).
3. *g*(*a*+*b*) ≤ *g*(*a*)∨*g*(*b*).
4. *g*(*r*.*a*) ≤ *g*(*a*).

Let *f*_*α*_, *g*_*α*_ : *M*⟶[0,1] be two composition functions, where *f*_*α*_=*αof* and *g*_*α*_=*αog*. Then, $\bar{A}=\left(M,f_{\alpha },g_{\alpha }\right)$ is called an *α*-intuitionistic fuzzy (*α*-IF) submodule if for *r* ∈ *R* and *x*, *y* ∈ *M* we have the following:

1. *f*_*α*_(*x*+*y*) ≥ *f*_*α*_(*x*)∧*f*_*α*_(*y*).
2. *f*_*α*_(*r*.*y*) ≥ *f*_*α*_(*x*).
3. *g*_*α*_(*x*+*y*) ≤ *g*_*α*_(*x*)∨*g*_*α*_(*y*).
4. *g*_*α*_(*r*.*y*) ≤ *g*_*α*_(*x*).

**Remark 1 .**

*IfA*=(*M*, *f*, *g*, *L*, *α*)*is an LIF-3 submodule, then*

(7) $$\begin{array}{c} f\left(0\right)\geq f\left(x\right),\\ g\left(0\right)\leq g\left(x\right). \end{array}$$

Moreover, if $\bar{A}=\left(M,f_{\alpha },g_{\alpha }\right)$ is an *α*-IF submodule, then

(8) $$\begin{array}{c} f_{\alpha }\left(0\right)\geq f_{\alpha }\left(x\right),\\ g_{\alpha }\left(0\right)\leq g_{\alpha }\left(x\right). \end{array}$$

**Proposition 1 .**

*LetA*=(*M*, *f*, *g*, *L*, *α*)*be an LIF-3 submodule. Then, the necessary and sufficient conditions forAto be anR-module are as follows:*

1. *f*(*k*_1_*x*+*k*_2_*y*) ≥ *f*(*x*)∧*f*(*y*),
2. *g*(*k*_1_*x*+*k*_2_*y*) ≤ *g*(*x*)∨*g*(*y*),

∀ *x*, *y* ∈ *M* and *k*_1_, *k*_2_ ∈ *R*.

**Definition 1 .**

*An LIF-3 submoduleA*=(*M*, *f*, *g*, *L*, *α*)*of a ringRis called an LIF-3 ideal if for eachx*, *y* ∈ *R*,

1. *f*(*x* − *y*) ≥ *f*(*x*)∧*f*(*y*).
2. *f*(*x*.*y*) ≥ *f*(*x*)∨*f*(*y*).
3. *g*(*x* − *y*) ≤ *g*(*x*)∨*g*(*y*).
4. *g*(*x*.*y*) ≥ *g*(*x*)∧*g*(*y*).

**Definition 2 .**

*LetA*=(*M*, *f*, *g*, *L*, *α*)*be an LIF-3 submodule; then, the level sets are defined as*

(9) $$\begin{array}{c} A_{l_{1}l_{2}}=\left\{m\in M:f\left(m\right)\geq l_{1},g\left(m\right)\leq l_{2}\right\}. \end{array}$$

In a similar fashion, we can define level sets for an *α* -IF submodule $\bar{A}=\left(M,f_{\alpha },g_{\alpha }\right)$ as follows:

(10) $$\begin{array}{c} {\bar{A}}_{t_{1}t_{2}}=\left\{m\in M:f_{\alpha }\left(m\right)\geq t_{1},g_{\alpha }\left(m\right)\leq t_{2}\right\}. \end{array}$$

We can also write that

(11) $$\begin{array}{c} f_{\alpha_{t_{1}}}=\left\{m\in M:f_{\alpha }\left(m\right)\geq t_{1}\right\},\\ g_{\alpha_{t_{2}}}=\left\{m\in M:g_{\alpha }\left(m\right)\geq t_{2}\right\}. \end{array}$$

### 3.1. LIF-3 Codes over Modules

If we consider a module *M*=*ℤ*_*p*^*k*^_^*n*^ which is a *ℤ*_*p*^*k*^_-module, then an LIF-3 submodule *A* of *M* is termed as an LIF-3 linear code having length *n* over *ℤ*_*p*^*k*^_.

**Proposition 2 .**

*LetA*=(*M*, *f*, *g*, *L*, *α*)*be an LIF-3 submodule and*$\bar{A}=\left(M,f_{\alpha },g_{\alpha }\right)$*be anα-IF submodule. LetA*_*l*_1_*l*_2__*and*${\bar{A}}_{t_{1}t_{2}}$*be the level sets forAand*$\bar{A}$*, whereα*(*l*_1_)=*t*_1_*andα*(*l*_2_)=*t*_2_*. Then,*$A_{l_{1}l_{2}}\subseteq {\bar{A}}_{t_{1}t_{2}}$.

**Proof**

Let *A*_*l*_1_*l*_2__ and ${\bar{A}}_{t_{1}t_{2}}$ be two level sets for *A* and $\bar{A}$. Let *m* ∈ *A*_*l*_1_*l*_2__; then, *f*(*m*) ≥ *l*_1_ and *g*(*m*) ≤ *l*_2_ imply that *f*_*α*_(*m*) ≥ *α*(*l*_1_) and *g*_*α*_(*m*) ≤ *α*(*l*_2_), where *α*(*l*_1_), *α*(*l*_2_) ∈ [0,1]. From this, we get that *m* ∈ *A*_*α*(*l*_1_)*α*(*l*_2_)_. Thus, *A*_*l*_1_*l*_2__⊆*A*_*α*(*l*_1_)*α*(*l*_2_)_. Let *α*(*l*_1_)=*t*_1_ and *α*(*l*_2_)=*t*_2_; then, *A*_*l*_1_*l*_2__⊆*A*_*t*_1_*t*_2__.

From the above proposition, we have concluded that the level set of LIF-3 submodule is contained in *α*-IF submodule, so we will use *α*-IF submodule for further discussion of codes. Let us consider a module *M*=*ℤ*_*p*^*k*^_^*n*^ which is a *ℤ*_*p*^*k*^_ module, and let $\bar{A}=\left(M,f_{\alpha },g_{\alpha }\right)$ be an *α*-IF submodule; then, $\bar{A}$ is said to be an *α*-IF linear code of length *n* over the module *ℤ*_*p*^*k*^_.

**Example 1 .**

*Considerℤ*_4_ *-module and latticeL*={0,1, *a*, *b*, *c*, *d*, }*having 0, 1 as bottom and top elements witha* ≤ *c*, *a* ≤ *d, andb* ≤ *c. Letf*, *g* : *ℤ*_4_⟶*Landα* : *L*⟶[0,1]*be defined as*

(12) $$\begin{array}{c} f=\left(\begin{array}{cccc} 0 & 1 & 2 & 3\\ 1 & a & b & c \end{array}\right),\\ g=\left(\begin{array}{cccc} 0 & 1 & 2 & 3\\ 0 & c & d & a \end{array}\right). \end{array}$$

Let *α*(0)=*α*(*b*)=0, *α*(*a*)=0.2=*α*(*c*), and *α*(*d*)=*α*(1)=1. Then, $\bar{A}=\left(M,f_{\alpha },g_{\alpha }\right)$ is an *α* -IF *ℤ*_4_ module; therefore, $\bar{A}$ is an *α* -IF linear code.

**Definition 3 .**

Consider an *α*-IF submodule $\bar{A}=\left(M,f_{\alpha },g_{\alpha }\right)$; then, the number of the elements mapped in to the same element, say, *t* ∈ [0,1], is said to be the degree of that element *t* and it is denoted by *p*_*t*_. In example 1, *p*_0_=2, *p*_1_=2, *p*_0.2_=2.

**Proposition 3 .**

*Consider anα-IF submodule* $\bar{A}=\left(M,f_{\alpha },g_{\alpha }\right)$ ; $\bar{A}$*is anα-IF linear code overℤ*_*p*^*k*^_*iff*${\bar{A}}_{t_{1}t_{2}}\neq \phi$*are linear codes.*

**Definition 4 .**

Let $\bar{A}$ be an *α*-IF submodule and *k* be non-empty subset of $\bar{A}$. The *α*-IF characteristic function of *k* is denoted by *χ*_*k*_=〈*f*_*αχ*_*k*__, *g*_*αχ*_*k*__〉, where *f*_*αχ*_*k*__, *g*_*αχ*_*k*__ : *M*⟶[0,1]; then,

(13) $$\begin{array}{c} f_{\alpha \chi_{k}}\left(x\right)=\left\{\begin{array}{cc} 1, & \text{if }x\in k;\\ 0, & \text{if }x\notin k, \end{array}\right.\\ g_{\alpha \chi_{k}}\left(x\right)=\left\{\begin{array}{cc} 0, & \text{if }x\in k;\\ 1, & \text{if }x\notin k. \end{array}\right. \end{array}$$

**Theorem 1 .**

*Let𝒞be a subset of anα-IF submoduleℤ*_*p* ^*k*^_^*n*^ *; then,𝒞is anα-IF linear code overℤ*_*p* ^*k*^_ *iffχ*_*C*_ *for𝒞is anα-IF linear code over the same ringℤ*_*p* ^*k*^_.

**Proof**

Let *𝒞* be a linear code implying that *𝒞* is an *α*-IF submodule. Now, if *x*, *y* ∈ *𝒞*, then by Definition 4 of *χ*_*C*_, we have

(14) $$\begin{array}{c} f_{\alpha \chi_{C}}\left(x\right)=1=f_{\alpha \chi_{C}}\left(y\right),\\ g_{\alpha \chi_{C}}\left(x\right)=0=g_{\alpha \chi_{C}}\left(y\right). \end{array}$$

As *𝒞* is an *α*-IF submodule, then *x*+*y*,  *r*.*x* ∈ *𝒞* and for all *r* ∈ *R* imply that *f*_*αχ*_*C*__(*x*+*y*)=1=1∧1=*f*_*αχ*_*C*__(*x*)∧*f*_*αχ*_*C*__(*y*) and *f*_*αχ*_*C*__(*r*.*x*)=1=*f*_*αχ*_*C*__(*x*). Similarly, *g*_*αχ*_*C*__(*x*+*y*) ≤ *g*_*αχ*_*C*__(*x*)∨*g*_*αχ*_*C*__(*y*) and *g*_*αχ*_*C*__(*r*.*x*) ≤ *g*_*αχ*_*C*__(*x*). Thus, the *χ*_*C*_ of *𝒞* is an *α*-IF submodule; hence, it is an *α*-IF linear code.

Conversely, suppose that the *χ*_*C*_ be a linear code; hence, it is an *α*-IF submodule. Let *x*, *y* ∈ *𝒞*; then, by Definition 4,

(15) $$\begin{array}{c} f_{\alpha \chi_{C}}\left(x\right)=1=f_{\alpha \chi_{C}}\left(y\right),\\ g_{\alpha \chi_{C}}\left(x\right)=0=g_{\alpha \chi_{C}}\left(x\right), \end{array}$$

imply that *f*_*αχ*_*C*__(*x*+*y*) ≥ *f*_*αχ*_*C*__(*x*)∧*f*_*αχ*_*C*__(*y*)=1∧1=1 and *f*_*αχ*_*C*__(*r*.*x*) ≥ *f*_*αχ*_*C*__(*x*)=1. Similarly, *g*_*αχ*_*C*__(*x*+*y*) ≤ *g*_*αχ*_*C*__(*x*)∨*g*_*αχ*_*C*__(*y*)=0∨0=0 and *g*_*αχ*_*C*__(*r*.*x*) ≥ *g*_*αχ*_*C*__(*x*)=0; hence, *x*+*y*,  *r*.*x* ∈ *𝒞*; thus, *𝒞* is an *α*-IF submodule, so *𝒞* is an *α*-IF linear code.

**Proposition 4 .**

*Let* $\bar{A}$ *be anα-IFS ofℤ*_*p* ^*k*^_^*n*^. $\bar{A}$*is anα-IF linear code overℤ*_*p*^*k*^_*iff*$\chi_{{\bar{A}}_{t_{1}t_{2}}}$*is anα-IF linear code.*

**Definition 5 .**

*Let* $\bar{A}=\left(M,f_{\alpha },g_{\alpha }\right)$ *be anα-IF fuzzy submodule; then,* $\bar{A}$ *is also a linear code such that fort*_1_, *t*_2_ ∈ [0,1], *f*_*α*_(*x*)=*t*_1_*andg*_*α*_(*x*)=*t*_2_*; then,Ais said to be a trivialα-IF linear code.*

**Definition 6 .**

*LetM*=*ℤ*_*p*^*k*^_^*n*^*be a module over the ringℤ*_*p*^*k*^_*. Then, twoα-IF submodules*$\bar{A}=\left(M,f_{\alpha },g_{\alpha }\right)$*and*$\bar{B}=\left(M,f_{\alpha }^{\prime },g_{\alpha }^{\prime }\right)$*are said to be orthogonal if*

(16) $$\begin{array}{c} \text{Im}\left(f_{\alpha }^{\prime }\right)=\left\{1-m:m\in \text{Im}\left(f_{\alpha }\right)\right\},\\ \text{Im}\left(g_{\alpha }^{\prime }\right)=\left\{1-m\prime :m\prime \in \text{Im}\left(g_{\alpha }\right)\right\}. \end{array}$$

Furthermore, for all *t*_1_, *t*_2_ ∈ [0,1], we have

(17) $$\begin{array}{c} f_{\alpha_{1-t_{1}}}={\left(f_{\alpha_{t_{1}}}\right)}^{T}\\ =\left\{b\in M|\langle a,b\rangle =0,\text{ for all a}\in f_{\alpha_{t_{1}}}\right\},\\ g_{\alpha_{1-t_{2}}}^{\prime }={\left(g_{\alpha_{t_{2}}}\right)}^{T}\\ =\left\{b\in M:\langle a,b\rangle =0\text{ for all }a\in g_{\alpha_{t_{2}}}\right\}\text{,} \end{array}$$

where 〈·, ·〉 is the inner product on module *M*.

**Example 2 .**

*Let* $\bar{A},\bar{B}:{\mathbb{Z}}_{4}⟶\left[0,1\right]$ *be twoα-IF submodules which are defined as*

(18) $$\begin{array}{c} f_{\alpha }=\left(\begin{array}{cccc} 0 & 1 & 2 & 3\\ 0.5 & 0.25 & 0.33 & 0.25 \end{array}\right),\\ g_{\alpha }=\left(\begin{array}{cccc} 0 & 1 & 2 & 3\\ 0.125 & 0.33 & 0.2 & 0.33 \end{array}\right)\text{,} \end{array}$$

and

(19) $$\begin{array}{c} f_{\alpha }^{\prime }=\left(\begin{array}{cccc} 0 & 1 & 2 & 3\\ 0.75 & 0.2 & 0.67 & 0.5 \end{array}\right),\\ g_{\alpha }^{\prime }=\left(\begin{array}{cccc} 0 & 1 & 2 & 3\\ 0.67 & 0.875 & 0.8 & 0.875 \end{array}\right). \end{array}$$

*Asf*_*α*_*t*_1___={*a* ∈ *M* : *f*_*α*_(*a*) ≥ *t*_1_}*, we can compute the valuesf*_*α*_*t*_1___*fort*_1_ ∈ [0,1].

(20) $$\begin{array}{c} f_{\alpha_{0.5}}=\left\{a\in M:f_{\alpha }\left(a\right)\geq 0.5\right\}\\ =\left\{0\right\},\\ f_{\alpha_{0.25}}=\left\{a\in M:f_{\alpha }\left(a\right)\geq 0.25\right\}\\ =\left\{0,1,2,3\right\}\\ ={\mathbb{Z}}_{4},\\ f_{\alpha_{0.33}}=\left\{a\in M:f_{\alpha }\left(a\right)\geq 0.33\right\}\\ =\left\{0,2\right\}. \end{array}$$

Similarly, the remaining values given in Table 1 and2 can be obtained from the definition of level set.

Hence, $\bar{A}$ is orthogonal to $\bar{B}$. We have shown the orthogonality of two sets, but under some conditions, the two sets are not orthogonal. This can be verified through the following remark.

**Remark 2 .**

*LetMbeℤ*_*p* ^*k*^_ *module and* $\bar{A}$ , $\bar{B}$*be twoα-IF submodules. If for allw* ∈ *M*,*f*_*α*_(*w*)=*rorg*_*α*_(*w*)=*r, wherer* ∈ [0,1]*, then set*$\bar{A}$*is not orthogonal to set*$\bar{B}$.

**Remark 3 .**

*Let* $\bar{A}=\left(M,f_{\alpha },g_{\alpha }\right)$ *be anα-IF submodule, so* $\bar{A}$ *is a linear code overℤ*_*p* ^*k*^_ *having lengthn. Consider sets*

(21) $$\begin{array}{c} \text{im}\left(f_{\alpha }\right)=\left\{f_{\alpha }\left(x\right)|x\in {\mathbb{Z}}_{p^{k}}^{n}\right\},\\ \text{im}\left(g_{\alpha }\right)=\left\{f_{\alpha }\left(x\right)|x\in {\mathbb{Z}}_{p^{k}}^{n}\right\}. \end{array}$$

*As the setℤ*_*p* ^*k*^_^*n*^ is finite, im(*f*_*α*_) and im(*g*_*α*_) are also finite. Consider an order *t*_1_ ≥ *t*_2_ ≥ ⋯≥*t*_*r*_; then, the set im(*f*_*α*_) satisfies this order. Similarly, suppose *t*_1_′ ≤ *t*_2_′ ≤ …≤*t*_*r*_′ and this order is satisfied by im(*g*_*α*_). ${\bar{A}}_{t_{1}t_{2}}$ is a level set which is a linear code, where the generator matrix for this linear code is given by *G*_*t*_*j*_*t*_*j*_′_ . Hence, *A* can be obtained from the matrices *G*_*t*_1_*t*_1_′_, *G*_*t*_2_*t*_2_′_,…, *G*_*t*_*r*_*t*_*r*_′_.

**Theorem 2 .**

*LetM*=*ℤ*_*p*^*k*^_^*n*^*be a finite module and*$\bar{A}=\left(M,f_{\alpha },g_{\alpha }\right)$*be anα-IF submodule ofM. Then, there is anα-IF submodule*$\bar{B}=\left(M,f_{\alpha }^{\prime },g_{\alpha }^{\prime }\right)$*such that*$\bar{A}$*is orthogonal to*$\bar{B}$*if and only if*|Im(*f*_*α*_)|, |Im(*g*_*α*_)| > 1*, and for anyr* ∈ Im(*f*_*α*_)*, there exists an elementη* ∈ Im(*f*_*α*_)*withf*_*αr*_=(*f*_*αη*_)^*T*^*. Similarly, for any elementp* ∈ Im(*g*_*α*_)*, there existð* ∈ Im(*g*_*α*_)*such thatg*_*αp*_=(*g*_*αð*_)^*T*^.

**Proof**

Let *M* be a module and $\bar{A}=\left(M,f_{\alpha },g_{\alpha }\right)$ be an *α*-IF submodule of *M*. Suppose that |Im(*f*_*α*_)|=*s* > 1 and |Im(*g*_*α*_)|=*k* > 1 and for any element *r* ∈ Im(*f*_*α*_) there is an element *η* ∈ Im(*f*_*α*_) such that *f*_*αr*_=(*f*_*αη*_)^*T*^. Moreover, for any element *p* ∈ Im(*g*_*α*_), there exists an element *ð* ∈ Im(*g*_*α*_) such that *g*_*αp*_=(*g*_*αð*_)^*T*^.Now, consider an order for Im(*f*_*α*_) and Im(*g*_*α*_) as

(22) $$\begin{array}{c} \text{Im}\left(f_{\alpha }\right)=\left(t_{1}\geq t_{2}\geq \cdots \geq t_{m}\right),\\ \text{Im}\left(g_{\alpha }\right)=\left(t_{1}^{\prime }\leq t_{2}^{\prime }\leq \cdots \leq t_{m}^{\prime }\right). \end{array}$$

By using these compositions, we can also define sets which form partition of module *M* as

(23) $$\begin{array}{c} M_{j}=\left\{a\in M:f_{\alpha }\left(a\right)=t_{j}\right\},\\ M_{j}=\left\{a\in M:g_{\alpha }\left(a\right)=t_{j}^{\prime }\right\}\text{,} \end{array}$$

where *j*=1,…, *m*. Now, if we define an *α*-IF set $\bar{B}=\left(M,f_{\alpha }^{\prime },g_{\alpha }^{\prime }\right)$, where *f*_*α*_′, *g*_*α*_′ : *M*⟶[0,1], then for *a* ∈ *M*, we have *f*_*α*_′(*a*)=1 − *t*_*m*−*j*+1_ and *g*_*α*_′(*a*)=1 − *t*_*m*−*j*+1_′.

As we know, Im(*f*_*α*_)={*t*_1_ ≥ *t*_2_ ≥ ⋯≥*t*_*m*_} imply that *f*_*αt*_1__⊆*f*_*αt*_2__⊆⋯⊆*f*_*αt*_*m*__. Similarly, for Im(*g*_*α*_)={*t*_1_′ ≤ *t*_2_′ ≤ ⋯∈≤*t*_*m*_′}, we have *g*_*αt*_*m*__⊆⋯⊆*g*_*αt*_2__⊆*g*_*αt*_*m*__. As for any element *r* ∈ Im(*f*_*α*_), there is an element *η* ∈ Im(*α*°*f*) such that *f*_*αr*_=(*α*°*f*_*η*_)^*T*^. Similarly, for any *p* ∈ Im(*g*_*α*_), there is an element *ð* ∈ Im(*g*_*α*_) such that *g*_*αp*_=(*g*_*αð*_)^*T*^, as in finite module there is a property related to orthogonality by which *f*_*αt*_*j*__=(*f*_*αt*_*m*−*j*+1__)^*T*^. Accordingly, we have *f*_*α*1−*t*_*m*−*j*+1__′={*a* ∈ *M* : *f*_*α*_′(*a*) ≥ 1 − *t*_*m*−*j*+1_}=*M*_*j*_UM_*j*−1_*U* ⋯ UM_1_=*f*_*αt*_*j*__=(*f*_*αt*_*m*−*j*+1__)^*T*^.

Furthermore, *g*_*αt*_*j*_′_=(*g*_*αt*_*m*−*j*+1_′_)^*T*^ and *g*_*α*1−*t*_*m*−*j*+1_′_′={*a* ∈ *M* : *g*_*α*_′(*a*) ≥ 1 − *t*_*m*−*j*+1_′}=*M*_1_*U* … UM_*j*−1_UM_*j*_=*g*_*αt*_*j*__=(*g*_*αt*_*m*−*j*+1_′_)^*T*^. Thus, $\bar{B}=\left(M,f_{\alpha }^{\prime },g_{\alpha }^{\prime }\right)$ is an *α*-IF submodule.

Conversely, suppose that $\bar{A}=\left(M,f_{\alpha },g_{\alpha }\right)$ and $\bar{B}=\left(M,f_{\alpha }^{\prime },g_{\alpha }^{\prime }\right)$ be two *α*-IF submodules such that these two sets are orthogonal to each other; then, we have |Im(*f*_*α*_)| > 1 and |Im(*g*_*α*_)| > 1. For all *t*, *t*′ ∈ [0,1], we also have *f*_*α*1−*t*_1__′=(*f*_*αt*_)^*T*^ and *g*_*α*1−*t*_1_′_′=(*g*_*αt*_′)^*T*^; then, for any element *r* ∈ Im(*f*_*α*_), there exist *η* ∈ Im(*f*_*α*_) such that *f*_*αr*_=(*f*_*αη*_)^*T*^, and for any element *p* ∈ Im(*g*_*α*_), there exist *ð* ∈ Im(*g*_*α*_) such that *g*_*αp*_=(*g*_*αð*_)^*T*^; this is due to the reason that im(*f*_*α*_)′={1 − *t* : *t* ∈ *f*_*α*_} and im(*g*_*α*_′)={1 − *t*′ : *t*′ ∈ *g*_*α*_}.

**Theorem 3 .**

*Suppose that* $\bar{A}=\left(M,f_{\alpha },g_{\alpha }\right)$ , $\bar{B}=\left(M,f_{\alpha }^{\prime },g_{\alpha }^{\prime }\right)$*, and*$\bar{C}=\left(M,f_{\alpha }^{\prime \prime },g_{\alpha }^{\prime \prime }\right)$*be threeα-IF fuzzy submodules of a moduleMsuch that*$\bar{A}$*is orthogonal to*$\bar{B}$*and*$\bar{B}$*is orthogonal to*$\bar{C}$*; then,*$\bar{B}=\bar{C}$.

**Proof**

Let $\bar{A}=\left(M,f_{\alpha },g_{\alpha }\right)$, $\bar{B}=\left(M,f_{\alpha }^{\prime },g_{\alpha }^{\prime }\right)$, and $\bar{C}=\left(M,f_{\alpha }^{\prime \prime },g_{\alpha }^{\prime \prime }\right)$ be three *α*-IF fuzzy submodules of a module *M* such that $\bar{A}$ is orthogonal to $\bar{B}$ and $\bar{B}$ is orthogonal to $\bar{C}$. Let *b* ∈ *f*_*α*_1−*t*__′, for *t* ∈ [0,1]. Then, for *a* ∈ *f*_*α*_*t*__, 〈*a*, *b*〉=0. $\bar{B}$ is orthogonal to $\bar{C}$, which implies that *b* ∈ *f*_*α*_1−*t*__^″^. Thus, *f*_*α*_1−*t*__′⊆*f*_*α*_1−*t*__^″^. Therefore, *f*_*α*_*t*__^″^⊆*f*_*α*_*t*__′. By following the same strategy, we get *f*_*α*_*t*__′⊆*f*_*α*_*t*__^″^. Thus, *f*_*α*_′=*f*_*α*_^″^. In a similar manner, we can show that *g*_*α*_′ =  *g*_*α*_^″^. Hence, $\bar{B}=\bar{C}$.

**Corollary 1 .**

*LetMbe a finiteℤ*_*p* ^*k*^_ *module and* $\bar{A}=\left(M,f_{\alpha },g_{\alpha }\right)$ *be anα-IF submodule ofM; if there existsα-IFS* $\bar{B}=\left(M,f_{\alpha }^{\prime },g_{\alpha }^{\prime }\right)$ *such that* $\bar{B}$ *is orthogonal to the submodule* $\bar{A}$ *, then* $\bar{B}$ *is anα-IF submodule of moduleM*.

**Definition 7 .**

*Consider twoα-IF linear codes* $\bar{A}=\left(M,f_{\alpha },g_{\alpha }\right)$ *and* $\bar{B}=\left(M,f_{\alpha }^{\prime },g_{\alpha }^{\prime }\right)$ *; then, these twoα-IF codes are said to be equivalent if the level sets* ${\bar{A}}_{t_{1}t_{2}}$ *and* ${\bar{B}}_{t_{1}t_{2}}$ *(which are also linear codes) for* $\bar{A}$ *and* $\bar{B}$ *are equivalent.*

### 3.2. *α*-IF Cyclic Codes

Cyclic codes have long since been one of the most interesting families of codes because of their rich algebraic structure, and these codes play an important role in coding theory. In this section, we will discuss *α*-IF cyclic codes and some important results related to these codes.

**Definition 8 .**

*Consider anα-IF submodule* $\bar{A}=\left(M,f_{\alpha },g_{\alpha }\right)$ *of moduleℤ*_*p* ^*k*^_^*n*^ *; then,* $\bar{A}$ *is called anα-IF cyclic code having lengthnoverℤ*_*p* ^*k*^_ *if for each*(*w*_0_, *w*_1_,…, *w*_*n*−1_) ∈ *ℤ*_*p*^*k*^_^*n*^*we have*

(24) $$\begin{array}{c} f_{\alpha }\left(w_{n-1},w_{0},\ldots ,w_{n-2}\right)\geq f_{\alpha }\left(w_{0},w_{1},\ldots ,w_{n-1}\right),\\ g_{\alpha }\left(w_{n-1},w_{0},\ldots ,w_{n-2}\right)\leq g_{\alpha }\left(w_{0},w_{1},\ldots ,w_{n-1}\right). \end{array}$$

**Proposition 5 .**

*Theα-IF submodule* $\bar{A}=\left(M,f_{\alpha },g_{\alpha }\right)$ *is anα-IF cyclic code overℤ*_*p* ^*k*^_ *if and only if* ${\bar{A}}_{t_{1}t_{2}}\neq \emptyset$ *are cyclic codes overℤ*_*p* ^*k*^_.

**Proof**

Let $\bar{A}=\left(M,f_{\alpha },g_{\alpha }\right)$ be an *α*-IF submodule and $\bar{A}$ be a cyclic code if ${\bar{A}}_{t_{1}t_{2}}$ is non-empty; then, for any $\left(w_{i}\right)\in {\bar{A}}_{t_{1}t_{2}}$, where *i*=0,1,…, *n* − 1 we have

(25) $$\begin{array}{c} f_{\alpha }\left(w_{0},w_{1},\ldots ,w_{n-1}\right)\geq t_{1},\\ g_{\alpha }\left(w_{0},w_{1},\ldots ,w_{n-1}\right)\leq t_{2}. \end{array}$$

As $\bar{A}$ is a cyclic code, we have

(26) $$\begin{array}{c} f_{\alpha }\left(w_{n-1},w_{0},\ldots ,w_{n-2}\right)\geq f_{\alpha }\left(w_{0},w_{1},\ldots ,w_{n-1}\right)\geq t_{1},\\ g_{\alpha }\left(w_{n-1},w_{0},\ldots ,w_{n-2}\right)\leq g_{\alpha }\left(w_{0},w_{1},\ldots ,w_{n-1}\right)\geq t_{2}\text{,} \end{array}$$

which imply that $\left(w_{n-1},w_{0},\ldots ,w_{n-2}\right)\in {\bar{A}}_{t_{1}t_{2}}$. Thus, ${\bar{A}}_{t_{1}t_{2}}$ is cyclic code.

Conversely, suppose that ${\bar{A}}_{t_{1}t_{2}}\neq \phi$ and ${\bar{A}}_{t_{1}t_{2}}$ is a cyclic code. If $\bar{A}$ is not a cyclic code, then there is an element (*w*_0_, *w*_1_,…, *w*_*n*−1_) ∈ *ℤ*_*p*^*k*^_^*n*^ such that *f*_*α*_(*w*_*n*−1_, *w*_0_,…, *w*_*n*−2_) < *f*_*α*_(*w*_0_, *w*_1_,…, *w*_*n*−1_). Suppose *t*_1_′=*f*_*α*_(*w*_0_, *w*_1_,…, *w*_*n*−1_). Similarly, *t*_2_′=*g*_*α*_(*w*_0_, *w*_1_,…, *w*_*n*−1_) imply that $\left(w_{0},w_{1},\ldots ,w_{n-1}\right)\in {\bar{A}}_{t_{1}^{\prime }t_{2}^{\prime }}$; thus, ${\bar{A}}_{t_{1}^{\prime }t_{2}^{\prime }}\neq \phi$ is a cyclic code but $\left(w_{n-1},w_{0},\ldots ,w_{n-2}\right)\notin {\bar{A}}_{t_{1}^{\prime }t_{2}^{\prime }}$, which is a contradiction, so $\bar{A}$ is a cyclic code.

**Proposition 6 .**

*Letℤ*_*p* ^*k*^_^*n*^ *be a module and* $\bar{A}=\left(M,f_{\alpha },g_{\alpha }\right)$ *be anα-IF submodule.* $\bar{A}$ *is anα-IF cyclic code on moduleℤ*_*p* ^*k*^_^*n*^ *if and only if the characteristic function of a level set* ${\bar{A}}_{t_{1}t_{2}}$ *is anα-IF cyclic code onℤ*_*p* ^*k*^_^*n*^.

**Proposition 7 .**

*Consider a moduleℤ*_*p* ^*k*^_^*n*^ *; then,* $\bar{A}=\left(M,f_{\alpha },g_{\alpha }\right)$ *is anα-IF cyclic code on moduleℤ*_*p* ^*k*^_^*n*^ *if and only if for each*(*w*_0_, *w*_1_,…, *w*_*n*−1_) ∈ *ℤ*_*p*^*k*^_^*n*^*we have*

(27) $$\begin{array}{c} f_{\alpha }\left(w_{0},w_{1},\ldots ,w_{n-1}\right)=f_{\alpha }\left(w_{n-1},w_{0},\ldots ,w_{n-2}\right)\\ =\cdots \\ =f_{\alpha }\left(w_{1},w_{2},\ldots ,w_{n-1},w_{0}\right),\\ g_{\alpha }\left(w_{0},w_{1},\ldots ,w_{n-1}\right)=g_{\alpha }\left(w_{n-1},w_{0},\ldots ,w_{n-2}\right)\\ =\cdots \\ =g_{\alpha }\left(w_{1},w_{2},\ldots ,w_{n-1},w_{0}\right). \end{array}$$

**Proof**

Let *ℤ*_*p*^*k*^_^*n*^ be a module and $\bar{A}=\left(M,f_{\alpha },g_{\alpha }\right)$ be an *α*-IF cyclic code on module *ℤ*_*p*^*k*^_^*n*^; then, we have

(28) $$\begin{array}{c} f_{\alpha }\left(w_{0},w_{1},\ldots ,w_{n-1}\right)\geq f_{\alpha }\left(w_{n-1},w_{0},\ldots ,w_{n-2}\right)\geq \cdots \geq f_{\alpha }\left(w_{1},w_{2},\ldots ,w_{n-1},w_{0}\right)\geq f_{\alpha }\left(w_{0},w_{1},\ldots ,w_{n-1}\right),\\ g_{\alpha }\left(w_{0},w_{1},\ldots ,w_{n-1}\right)\leq g_{\alpha }\left(w_{n-1},w_{0},\ldots ,w_{n-2}\right)\leq \cdots \leq g_{\alpha }\left(w_{1},w_{2},\ldots ,w_{n-1},w_{0}\right)\leq g_{\alpha }\left(w_{0},w_{1},\ldots ,w_{n-1}\right). \end{array}$$

Thus,

(29) $$\begin{array}{c} f_{\alpha }\left(w_{0},w_{1},\ldots ,w_{n-1}\right)=f_{\alpha }\left(w_{n-1},w_{0},\ldots ,w_{n-2}\right)\\ =\cdots \\ =f_{\alpha }\left(w_{1},w_{2},\ldots ,w_{n-1},w_{0}\right)\\ =f_{\alpha }\left(w_{0},w_{1},\ldots ,w_{n-1}\right),\\ g_{\alpha }\left(w_{0},w_{1},\ldots ,w_{n-1}\right)=g_{\alpha }\left(w_{n-1},w_{0},\ldots ,w_{n-2}\right)\\ =\cdots \\ =g_{\alpha }\left(x_{1},w_{2},\ldots ,w_{n-1},w_{0}\right)\\ =g_{\alpha }\left(w_{0},w_{1},\ldots ,w_{n-1}\right). \end{array}$$

Conversely, suppose that the above equality holds for *f*_*α*_ and *g*_*α*_; then, by Definition 8, *A* is an *α*-IF cyclic code.

**Theorem 4 .**

*Consider twoα-IF cyclic codes* $\bar{A}$ *and* $\bar{B}$ *; then, we have the following:*

1.
$\bar{A}\cap \bar{B}$ *is anα-IF cyclic code*.
2.
$\bar{A}+\bar{B}$ *is anα-IF cyclic code.*
3.
$\bar{A}\bar{B}$ *is anα-IF cyclic code.*

**Proof**

(1) Let $\bar{A}=\left(M,f_{\alpha },g_{\alpha }\right)$ and $\bar{B}=\left(M,f_{\alpha }^{\prime },g_{\alpha }^{\prime }\right)$ be two *α*-IF modules of module *ℤ*_*p*^*k*^_^*n*^ such that $\bar{A}$ and $\bar{B}$ are *α*-IF cyclic codes corresponding to *α*-IF modules. Then, for (*w*_0_, *w*_1_,…, *w*_*n*−1_) ∈ *ℤ*_*p*^*k*^_^*n*^,
  (30) $$\begin{array}{c} f_{\alpha }\cap f_{\alpha }^{\prime }\left(\left(w_{n-1},w_{0},\ldots ,w_{n-2}\right)\right)=\min\left\{\begin{array}{c} f_{\alpha }\left(\left(w_{n-1},w_{0},\ldots ,w_{n-2}\right)\right),\\ f_{\alpha }^{\prime }\left(\left(w_{n-1},w_{0},\ldots ,w_{n-2}\right)\right) \end{array}\right\}\geq \min\left\{f_{\alpha }\left(\left(w_{0},w_{1},\ldots ,w_{n-1}\right)\right),f_{\alpha }^{\prime }\left(\left(w_{0},w_{1},\ldots ,w_{n-1}\right)\right)\right\}\\ =f_{\alpha }\left(\left(w_{0},w_{1},\ldots ,w_{n-1}\right)\right)\cap f_{\alpha }^{\prime }\left(\left(w_{0},w_{1},\ldots ,w_{n-1}\right)\right). \end{array}$$
In a similar manner, we have
  (31) $$\begin{array}{c} g_{\alpha }\cap g_{\alpha }^{\prime }\left(\left(w_{0},w_{1},\ldots ,w_{n-1}\right)\right)\leq g_{\alpha }\left(\left(w_{0},w_{1},\ldots ,w_{n-1}\right)\right)\cap g_{\alpha }^{\prime }\left(\left(w_{0},w_{1},\ldots ,w_{n-1}\right)\right). \end{array}$$

 By taking intersection of two *α*-IF modules, we get again an *α*-IF module; thus, $\bar{A}\cap \bar{B}$ is an *α*-IF cyclic code.

(2) Now, for (*w*_0_, *w*_1_,…, *w*_*n*−1_) ∈ *ℤ*_*p*^*k*^_^*n*^,
  (32) $$\begin{array}{c} \left(f_{\alpha }+f_{\alpha }^{\prime }\right)\left(w_{n-1},\ldots ,w_{n-2}\right)=\max_{j}\left\{{\text{min}}_{y_{j}+z_{j}=w_{j}}\left\{f_{\alpha }\left(y_{n-1},\ldots ,y_{n-2}\right),f_{\alpha }^{\prime }\left(z_{n-1},\ldots ,z_{n-2}\right)\right\}\right\}\\ \geq \max\left\{\min\left\{f_{\alpha }\left(y_{0},y_{1},\ldots ,y_{n-1}\right),f_{\alpha }^{\prime }\left(z_{n-1},z_{0},\ldots ,z_{n-1}\right)\right\}\right\}\\ =\left(f_{\alpha }+f_{\alpha }^{\prime }\right)\left(\left(w_{0},w_{1},\ldots ,w_{n-1}\right)\right). \end{array}$$
Similarly, we can show (*g*_*α*_+*g*_*α*_′)(*w*_*n*−1_, *w*_0_,…, *w*_*n*−2_) ≤ (*g*_*α*_+*g*_*α*_′)(*w*_0_, *w*_1_,…, *w*_*n*−1_). Thus, $\bar{A}+\bar{B}$ is an *α*-IF cyclic code.
(3) Proof follows from (2).

**Proposition 8 .**

*Ifℤ*_*p* ^*k*^_^*n*^ *is a module, then* $\bar{A}$ *is said to be anα-IF cyclic code* iff *the non-empty level sets*${\bar{A}}_{t_{1}t_{2}}$*areα-IF ideals of the factor ring*(*ℤ*_*p*^*k*^_[*X*]/(*X*^*n*^ − 1)).

**Proof**

Consider a module *ℤ*_*p*^*k*^_^*n*^ and a factor ring (*ℤ*_*p*^*k*^_[*X*]/(*X*^*n*^ − 1)). Define a mapping as *ϕ* : *ℤ*_*p*^*k*^_^*n*^⟶(*Z*_*p*^*k*^_[*X*]/(*X*^*n*^ − 1)) which is also an isomorphism. Let *b*=(*b*_0_, *b*_1_,…, *b*_*n*−1_) ∈ *ℤ*_*p*^*k*^_^*n*^ such that *ϕ*(*b*)=∑_*j*=0_^*m*−1^*b*_*j*_*X*^*j*^.

Suppose that $\bar{A}$ be an *α*-IF cyclic code; this implies that ${\bar{A}}_{t_{1}t_{2}}\neq \emptyset$ is an *α*-IF cyclic code over *ℤ*_*p*^*k*^_. Cyclic codes are ideals in factor ring, which implies that ${\bar{A}}_{t_{1}t_{2}}$ is ideal of factor ring.

Conversely, suppose that for *t*_1_, *t*_2_ ∈ [0,1], the set ${\bar{A}}_{t_{1}t_{2}}$ is non-empty; ${\bar{A}}_{t_{1}t_{2}}$ being an ideal of a factor ring implies that ${\bar{A}}_{t_{1}t_{2}}$ is a submodule. Thus, the level set ${\bar{A}}_{t_{1}t_{2}}$ is a linear code implying the linearity of $\bar{A}$. If we define mapping *ϕ*, then the level set ${\bar{A}}_{t_{1}t_{2}}$ is an *α*-IF cyclic code; thus, $\bar{A}$ is an *α*-IF cyclic code over ring *ℤ*_*p*^*k*^_.

As *ℤ*_*p*^*k*^_ is a finite ring and if $\bar{A}=\left(M,f_{\alpha },g_{\alpha }\right)$ is a submodule, then im(*f*_*α*_) and im(*g*_*α*_) are also finite; then, we have ${\bar{A}}_{t_{1}t_{1}^{\prime }}\subseteq {\bar{A}}_{t_{2}t_{2}^{\prime }}\subseteq \cdots \subseteq {\bar{A}}_{t_{r-1}t_{r-1}^{\prime }}\subseteq {\bar{A}}_{t_{2}t_{2}^{\prime }}={\mathbb{Z}}_{p^{k}}^{n}$. Suppose *g*_*j*_^*k*^(*X*) ∈ *ℤ*_*p*^*k*^_[*X*] is the generator polynomial for ${\bar{A}}_{t_{i}t_{i}}^{\prime }$; then, *g*_*j*+1_^*k*^(*X*)/*g*_*j*_^*k*^(*X*), *j*=1,…, *r* − 1.

**Theorem 5 .**

*LetS*={*g*_1_^*k*^(*X*), *g*_2_^*k*^(*X*),…, *g*_*r*_^*k*^(*X*)}*be a set of polynomials* such that the polynomial *g*_*j*_(*X*) divides *X*^*n*^ − 1 for each *j*=1,…, *r. Ifg*_*j*+1_^*k*^(*X*)*|g*_*j*_^*k*^(*X*)*forj*=1,2,…, *r* − 1*andℤ*_*p*^*k*^_^*n*^=*g*_*j*+1_^*k*^(*X*)*, then the set of polynomials determines anα-IF cyclic code*$\bar{A}$*, whereg*_*j*_^*k*^(*X*)*is the collection of level cut cyclic subcodes of*$\bar{A}$.

**Proof**

Proof follows from Proposition 8.

### 3.3. IF Gray Map

The gray code, which is also called reflected binary code, is an ordering of the binary numeral system such that two successive values vary in a single bit. We will define *α*-IF gray code by using the compositions *f*_*α*_ and *g*_*α*_. Consider a map *η* : *ℤ*_2^2^_⟶*ℤ*_2_^2^; then, *η* is called a gray map defined as *η*(0)=00, *η*(1)=01, *η*(2)=11, *η*(3)=10.

**Definition 9 .**

*Consider a mappingη* : *ℤ*_2^2^_⟶*ℤ*_2_^2^*which is a gray map. Suppose thatS*(*ℤ*_2^2^_)*andS*(*ℤ*_2_^2^)*be twoα-IF fuzzy subsets ofℤ*_2_2__*andℤ*_2_^2^*. Forf*_*α*_, *g*_*α*_ ∈ *S*(*ℤ*_2^2^_)*, anα-IF gray mapη*^*∗*^ : *S*(*ℤ*_2^2^_)⟶*S*(*ℤ*_2_^2^)*is defined as*

(33) $$\begin{array}{c} \eta^{*}\left(f_{\alpha }\wedge g_{\alpha }\right)\left(b\right)=\text{sup}\left\{\left.f_{\alpha }\wedge g_{\alpha }\right)a|b=\eta \left(a\right)\right\}. \end{array}$$

**Example 3 .**

*Consider two mappingsf*_*α*_,  *g*_*α*_ : *ℤ*_4_⟶[0,1]*defined as follows:*

(34) $$\begin{array}{c} f_{\alpha }=\left(\begin{array}{cccc} 0 & 1 & 2 & 3\\ 1 & 0.4 & 0.4 & 0.4 \end{array}\right),\\ g_{\alpha }=\left(\begin{array}{cccc} 0 & 1 & 2 & 3\\ 0 & 0.3 & 0.3 & 0.3 \end{array}\right). \end{array}$$

By definition, we have

(35) $$\begin{array}{c} \eta^{*}\left(f_{\alpha }\wedge g_{\alpha }\right)\left(b\right)=\text{sup}\left\{\left.f_{\alpha }\wedge g_{\alpha }\right)a|b=\eta \left(a\right)\right\}. \end{array}$$

Now, for *a*=0,

(36) $$\begin{array}{c} \eta^{*}\left(f_{\alpha }\wedge g_{\alpha }\right)\left(b\right)=\text{sup}\left\{\left.f_{\alpha }\wedge g_{\alpha }\right)0|b=\eta \left(0\right)\right\}\\ \eta^{*}\left(f_{\alpha }\wedge g_{\alpha }\right)\left(00\right)=\text{sup}\left\{\left.f_{\alpha }\wedge g_{\alpha }\right)0|00=\eta \left(0\right)\right\}\\ =\text{sup}\left\{f_{\alpha }\left(0\right)\wedge g_{\alpha }\left(0\right)|00=\eta \left(0\right)\right\}\\ =\text{sup}\left\{1\wedge 0\right\}\\ =0. \end{array}$$

The values for *α*-IF gray map corresponding to these mappings are given in Table 3.

Now, we consider generalizing fuzzy gray map which is a mapping from *ℤ*_*p*^*k*^_ to *ℤ*_*p*_^*p*^*k*−1^^. Using this map, an *α*-IF generalized gray map can be defined.

**Definition 10 .**

*A mappingη* ^*∗*^ : *S*(*ℤ*_*p*^*k*^_)⟶*S*(*ℤ*_*p*_^*p*^*k*−1^^)*is said to be anα-IF generalized gray map if for anyf*_*α*_,  *g*_*α*_ ∈ *S*(*ℤ*_*p*^*k*^_)*we have*

(37) $$\begin{array}{c} \eta^{*}\left(f_{\alpha }\wedge g_{\alpha }\right)\left(b\right)=\left\{\begin{array}{cc} \left(f_{\alpha }\wedge g_{\alpha }\right)a, & \text{if }b=\eta \left(a\right);\\ 0, & \text{otherwise.} \end{array}\right. \end{array}$$

**Definition 11 .**

*If* $\bar{A}$ *is anα-IF code overℤ*_*p* ^*k*^_ *, then it is said to be anα-IFℤ*_*p* ^*k*^_ *-linear code if it is an image under theα-IF generalized gray map of a linear code overℤ*_*p* ^*k*^_.

**Definition 12 .**

*Consider anα-IF codeC; then, it is anα-IFℤ*_*p* ^*k*^_ *cyclic code ifCis anα-IFℤ*_*p* ^*k*^_ *-linear code and also if it is the image under theα-IF generalized gray map of a cyclic code overℤ*_*p* ^*k*^_.

**Example 4 .**

*Consider mappingsf*_*α*_, *g*_*α*_ : *ℤ*_2_^6^⟶[0,1]*where*

(38) $$\begin{array}{c} f_{\alpha }\left(x_{0},x_{1},x_{2},x_{3},x_{4},x_{5}\right)=\left\{\begin{array}{cc} 1, & \text{if }x_{4}=x_{5}=0;\\ 0, & \text{otherwise,} \end{array}\right. \end{array}$$

and

(39) $$\begin{array}{c} g_{\alpha }\left(x_{0},x_{1},x_{2},x_{3},x_{4},x_{5}\right)=\left\{\begin{array}{cc} 0, & \text{if }x_{4}=x_{5}=0;\\ 1, & \text{otherwise.} \end{array}\right. \end{array}$$

*Then,C*=(*M*, *f*_*α*_, *g*_*α*_) is an *α* -IF linear code having length six over *ℤ*_2_ . Now, if we consider another code where *f*_*α*_,  *g*_*α*_ : *ℤ*_4_^3^⟶[0,1] defined as$f_{\alpha }^{\prime }\left(x_{0},x_{1},x_{2}\right)=\left\{\begin{array}{cc} 1, & \text{if }x_{2}=0;\\ 0, & \text{otherwise,} \end{array}\right.$ and $g_{\alpha }^{\prime }\left(x_{0},x_{1},x_{2}\right)=\left\{\begin{array}{cc} 0, & \text{if }x_{2}=0;\\ 1, & \text{otherwise;} \end{array}\right.$ then, *C*=(*M*, *f*_*α*_, *g*_*α*_) is an *α* -IF linear code having length 3 over *ℤ*_4_.

**Theorem 6 .**

*Anα-IF gray mapη* ^*∗*^ *is a bijective map.*

**Proof**

This is due to the fact that *η*^*∗*^ is a one-to-one function.

### 3.4. Error Correction

The coding theory deals with encoding and decoding a message. During transmission of data, errors may occur; they can be detected and corrected by using different procedures such as parity check, syndrome decoding, and redundancy check which are for ordinary codes. Similarly, a method can be adopted in fuzzy codes for the confirmation about the codeword, that is, whether it belongs to the transmitted code or not. This can be done by considering the level set from which one can determine the confirmation degree as to whether a received codeword belongs to the original code. This method can be explained through the following example.

**Example 5 .**

*Consider a moduleℤ*_2_^4^ *, where*

(40) $$\begin{array}{c} {\mathbb{Z}}_{2}^{4}=\left\{0000,1000,0100,0010,0001,1100,1010,1001,0101,0110,0011,1011,1101,1110,0111,111\right\}. \end{array}$$

Let $\bar{A}=\left(M,f_{\alpha },g_{\alpha }\right)$ be an *α*-IF submodule where *f*_*α*_, *g*_*α*_ : *ℤ*_2_^4^⟶[0,1] and

(41) $$\begin{array}{c} C=\left\{0000,0001,0010,1100,0011,1101,1110,1111\right\}, \end{array}$$

*which is a subset ofℤ*_2_^4^ *and also a linear code. Now, by transmitting this code, the received codeword has errors; assume that the received codewords are*{000; .01, 010; 0.001, 011; 0.01, 100; 0.1, 101; 0.01, 110; \\0.1, 001; 0.01, 111; 0.999}.

Table 4 shows the values of *f*_*α*_, *g*_*α*_ corresponding to the codewords of code *C*.

The level set is defined as ${\bar{A}}_{t_{1}t_{2}}=\left\{a\in M:\alpha \text{ of }\left(a\right)\geq t_{1},\alpha og\left(a\right)\leq t_{2}\right\}$; from this, we get for *t*_1_ > 0.9 and *t*_2_ ≤ 0.01 that the received codeword is in *C*.

### 3.5. Application

#### 3.5.1. Sphere in *α*-IF Linear Code

Suppose *ℤ*_*p*^*k*^_^*m*^ be a module and $\bar{A}=\left(M,f_{\alpha },g_{\alpha }\right)$ be an *α*-IF submodule. If *𝒞* is an *α*-IF linear code, then the level set *𝒞*_*t*_1_*t*_2__ of *𝒞* is also an *α*-IF linear code. As the elements in *ℤ*_*p*^*k*^_^*m*^ are the words of length *m* over the alphabet {0,1,2,…, *p*^*k*^ − 1} and the level set *𝒞*_*t*_1_*t*_2__ also consists of the elements of *ℤ*_*p*^*k*^_^*m*^, then for a member *a* ∈ *𝒞*_*t*_1_*t*_2__ and for any integer *r* ≥ 0, the sphere of radius *r* and center *a* is defined as follows.

**Theorem 7 .**

*Consider a moduleM*=*ℤ*_*p*^*k*^_^*m*^; *then*, *the sphere having radiusr*(where 0 ≤ *r* ≤ *m*))*contains*

(42) $$\begin{array}{c} \left(\begin{array}{c} m\\ 0 \end{array}\right)+\left(\begin{array}{c} m\\ 1 \end{array}\right)\left(p^{k}-1\right)+\left(\begin{array}{c} m\\ 2 \end{array}\right){\left(p^{k}-1\right)}^{2}+\cdots +\left(\begin{array}{c} m\\ r \end{array}\right){\left(p^{k}-1\right)}^{r}, \end{array}$$

words.

**Proof**

Consider a module *ℤ*_*p*^*k*^_^*m*^. Let *x* be a fixed word in *ℤ*_*p*^*k*^_^*m*^. Then, the number of the words which differ from *x* in *m* position is $\left(\begin{array}{c} m\\ w \end{array}\right){\left(p^{k}-1\right)}^{w}$.

**Example 6 .**

*LetM*=*ℤ*_2_^2^*, whereℤ*_2_^2^={00,01,10,11}*. Here,k*=1, *p*=2, *m*=2*, and*0 ≤ *r* ≤ 2*. If we take a wordx*=10*, then its distances from the remaining codewords ared*(10,00)=1, *d*(10,01)=2, *d*(10,11)=1*, and the sum of these distances is equal to 4. Now,*$\left(\begin{array}{c} 2\\ 0 \end{array}\right)+\left(\begin{array}{c} 2\\ 1 \end{array}\right)\left(2-1\right)+\left(\begin{array}{c} 2\\ 2 \end{array}\right){\left(2-1\right)}^{2}=1+2+1=4$*, which is also equal to 4.*

### 3.6. Genetic Code with Module *ℤ*_64_

Amino acid sequence of a protein can be determined from the sequence or order of DNA and RNA molecules [26]. Although the information about protein sequence is found in the sequence of nucleotides in DNA, we cannot say that the proteins formed directly from the DNA. RNA molecules are also involved in the formation of proteins. RNA is composed of four bases which are adenine (A), guanine (G), cytosine (C), and uracil (U). One unit is formed from the three adjacent bases which are called a codon which codes for an amino acid. For instance, the amino acid methionine is encoded by the codon AUG. There are 64 codons; 20 amino acids are specified by the 61 codons that make up proteins. The remaining three codons do not code for any amino acid.

There is another important aspect related to genetic code which is the algebraic structure of a genetic code from which some important information can be obtained [27]. Many scientists [28, 29] have studied the algebraic structure of codons. They explore various methods to model the genetic code mathematically and use binary representation for the four bases. Nemzer [30] established binary representation of genetic codes. Press et al. [31] described the HEDGES (Hash Encoded, Decoded by Greedy Exhaustive Search) error-correcting code to repair basic DNA errors. Rocha et al. [32] analyzed the DNA sequence generated by linear codes over the ring *ℤ*_4_. Bennenni et al. [33], Dinh et al. [34], and Gowthaman et al. [35] investigated DNA cyclic codes over rings.

In the presented study, codon structure is investigated by involving the lattice structure of codons along with module over a ring. As there are 64 possible codons, we take *ℤ*_64_ module. From this, we conclude that *α*-IF submodule with module *ℤ*_64_ is a linear code. Consider a module *M*=*ℤ*_64_ and let *f*_*α*_, *g*_*α*_ : *ℤ*_64_⟶[0,1]. Suppose *f*, *g* : *ℤ*_64_⟶*L* where *L* is a lattice consisting of 64 codons; the values for these functions and their composition are shown in Table 5. Here, $\bar{A}=\left(M,f_{\alpha },g_{\alpha }\right)$ is an *α*-IF submodule so it is an *α*-IF linear code. We will use the following methodology.

Consider the lattice *L* comprises 64 codons and a module *Z*_64_={0,1,…, 63} which also consists of 64 elements. Let *a*=0 ∈ *Z*_64_; we know that *f*, *g* : *Z*_64_⟶*L*. Then, *f*(0)=*UUU*, *g*(0)=*AAA*, which are top and bottom elements of the lattice *L* as shown in [36]. As *f*_*α*_, *g*_*α*_ : *ℤ*_64_⟶[0,1], assign the values to these functions as *f*_*α*_(0)=1 ∈ [0,1], *g*_*α*_(0)=0 ∈ [0,1], where *f*_*α*_(0)+*g*_*α*_(0) ≤ 1. Similarly, values can be assigned to the remaining codons, which is shown in Table 5. Then, by Definition 3, *α*-IF submodule with module *Z*_64_ is referred to as a linear code. By using Definition 4, we can compute the degrees of element *t* ∈ [0,1]; for example, for *t*=1, the degree is 1 and for *t*=0.3 the degree is 10. The remaining values can be computed in a similar way. Degrees of respective codons are also given in Table 5.

The most important properties of amino acids are their hydrophobic and hydrophilic properties. As in lattice diagram, codons with second base *U* show hydrophobic amino acids, and those with second base *A* show hydrophilic amino acids. There are total 16 codons that specify hydrophobic and hydrophilic amino acids, so we can define an *α*-IF linear code by considering the *ℤ*_16_ module. Let *f*, *g* : *ℤ*_16_⟶*L* and *f*_*α*_, *g*_*α*_ : *ℤ*_16_⟶[0,1], where lattice *L* consists of 16 elements. A comparison can be made between the two properties of amino acid by employing the concept of degree given in Definition 4. It can be seen in Table 6 that the average degree of hydrophobic amino acids is greater than the hydrophilic amino acids.

## 4. Conclusion

Communication systems are designed for data transmission, working on the principle of encoding and decoding information. In classical coding theory, different procedures can be adopted to detect and correct errors that may arise during communication. The communication process is highly influenced by vagueness, inaccuracy, imprecision, and uncertainty. The classical coding methodologies are sometimes not efficient in handling such situations. Therefore, fuzzy logic is a viable option to handle such type of information. Lattice valued intuitionistic fuzzy sets are the generalization of basic fuzzy sets that incorporated the degree of both membership and nonmembership. Thus, they constitute a more efficient framework to model uncertain data. In this article, LIF-3 submodule and *α*-IF submodule are defined. These two structures are related by means of their level sets; that is, the level set of LIF-3 submodule is contained in the level set of *α*-IF submodule. After obtaining this result, further codes are discussed over *α*-IF submodule. Linear codes and cyclic codes are defined over *α*-IF submodule. Some important properties and results related to these codes are investigated. It is also concluded that this concept of *α*-IF linear codes is entirely applicable in genetic code. There are 64 codons that specify different amino acids, so *Z*_64_ module is considered here and *α*-IF linear codes are defined over *Z*_64_ module.

In literature, conventional codes such as hamming codes and Hadamard codes are defined, which are constructed by considering vector spaces and fields. Modules are the extension of vector spaces. Thus, this work can be extended further by employing the present methodology and by using modules instead of vector spaces and fields. This can be done by investigating these codes over *α*-IF submodule by involving *ℤ*_*p*^*k*^_^*n*^ module over a ring *ℤ*_*p*^*k*^_ instead of the field *𝔽*. Moreover, there are several generalizations of fuzzy sets like picture fuzzy sets [37], Pythagorean fuzzy sets [38], hesitant fuzzy sets [39], and neutrosophic sets [40] where the concept of lattices and codes can be extended to cope with the uncertainty in a better way. In “Application,” a link is made by incorporating the genetic code along with the concept of lattice valued intuitionistic fuzzy sets of type-3, and *α*-IF codes are defined by considering the modules *Z*_64_ and *Z*_16_. This methodology seems to be attractive for further investigation of genetic codes.

## Figures and Tables

**Table 1:** Values of orthogonal set for membership.

*t*_1_	*f*_*α*_*t*_1___	*f*_*α*_1−*t*_1___	(*f*_*α*_*t*_1___)^*T*^
0.5	{0}	*ℤ*_4_	*ℤ*_4_
0.25	*ℤ*_4_	{0}	{0}
0.33	{0,2}	{0,2}	{0,2}
0.25	*ℤ*_4_	{0}	{0}
			
			
			
			
			

**Table 2:** Values of orthogonal set for nonmembership.

*t*_2_	*g*_*α*_*t*_2___	*g*_*α*_1−*t*_2___	(*g*_*α*_*t*_2___)^*T*^
0.125	{0}	*ℤ*_4_	*ℤ*_4_
0.33	*ℤ*_4_	{0}	{0}
0.2	{0,2}	{0,2}	{0,2}
0.33	*ℤ*_4_	{0}	{0}

**Table 3:** Values of gray map and *α*-IF gray map.

(*η*(*a*)=*b*)	(*η*^*∗*^(*f*_*α*_∧*g*_*α*_)(*b*))
00	0
01	0.3
11	0.3
10	0.3

**Table 4:** Recovering of codewords

*a*	*f*_*α*_	*g*_*α*_	*a*	*f*_*α*_	*g*_*α*_	*a*	*f*_*α*_	*g*_*α*_	*a*	*f*_*α*_	*g*_*α*_
0000	1	0	1000	0.9	0.1	0100	0.9	0.1	0010	0.99	0.01
0001	0.99	0.01	1100	0.99	0.01	1010	0.9	0.1	1001	0.9	0.1
0101	0.9	0.1	0110	0.9	0.1	0011	0.99	0.01	1011	0.9	0.1
1101	0.99	0.01	1110	0.99	0.01	0111	0.9	0.1	1111	0.99	0.01

**Table 5:** Codons along with their respective degrees.

*a*	*f*(*a*)	*g*(*a*)	*a*(*f*(*a*))
0	UUU	AAA	1
1	UCU	AGA	0.35
2	UUG	CAA	0.4
3	UCG	AGC	0.3
4	GGU	AAC	0.4
5	GCU	CGA	0.3
6	UUC	GAA	0.35
7	UCC	AGG	0.28
8	GUG	CAC	0.35
9	GCG	CGC	0.28
10	CUU	AAG	0.35
11	CCU	GGA	0.28
12	UUA	UAA	0.3
13	UCA	AGU	0.28
14	GUC	GAC	0.3
15	GCC	CGG	0.25
16	CUG	CAG	0.3
17	CCG	GGC	0.25
18	AUU	AAU	0.3
19	ACU	UGA	0.25
20	GUA	UAC	0.28
21	GCA	CGU	0.2
22	CUC	GAG	0.28
23	CCC	GGG	0.2
24	AUG	CAU	0.28
25	ACG	UGC	0.2
26	CUA	UAG	0.25
27	CCA	GGU	0.35
28	AUC	GAU	0.25
29	ACC	UGG	0.15
30	AUA	UAU	0.25
31	ACA	UGU	0.1
32	UGU	ACA	0.4
33	UAU	AUA	0.3
34	UGG	ACC	0.35
35	UAG	AUC	0.28
36	GGU	CCA	0.35
37	GAU	CUA	0.28
38	UGC	ACG	0.3
39	UAC	AUG	0.25
40	GGG	CCC	0.3
41	GAG	CUC	0.25
42	CGU	GCA	0.3
43	CAU	GUA	0.25
44	UGA	ACU	0.28
45	UAA	AUU	0.28
46	GGC	CCG	0.28
47	GAC	CUG	0.28
48	CGG	GCC	0.28
49	CAG	GUC	0.28
50	AGU	UCA	0.28
51	AAU	UUA	0.28
52	GGA	CCU	0.25
53	GAA	CUU	0.2
54	CGC	GCG	0.25
55	CAC	GUG	0.2
56	AGG	UCC	0.25
57	AAG	UUC	0.2
58	CGA	GCU	0.2
59	CAA	GUU	0.15
60	AGC	UCG	0.2
61	ACC	UUG	0.15
62	AGA	UCU	0.15
63	AAA	UUU	0

**Table 6:** Codons with *U* or *A* as a second base which are coding for hydrophobic or hydrophilic amino acid.

*a*	*f*(*a*)	*f*_*α*_(*a*)	Degree	*g*(*a*)	*g*_*α*_(*a*)	Degree	*a*	*f*(*a*)	*f*_*α*_(*a*)	Degree	*g*(*a*)	*g*_*α*_(*a*)	Degree
0	UUU	1	1	AAA	0	1	1	AUA	0.1	1	AAC	0.01	2
2	AUC	0.12	2	CAA	0.01	2	3	CUA	0.12	2	AAG	0.05	3
4	AUG	0.2	3	CAC	0.05	3	5	CUC	0.2	3	GAA	0.05	3
6	GUA	0.2	3	AAU	0.1	4	7	AUU	0.25	4	CAG	0.1	4
8	CUG	0.25	4	GAC	0.1	4	9	GUC	0.25	4	UAA	0.1	4
10	UUA	0.25	4	CAU	0.12	2	11	CUU	0.3	3	GAG	0.12	2
12	GUG	0.3	3	UAC	0.12	2	13	UUC	0.3	3	GAU	0.2	2
14	GUU	0.35	2	UAG	0.2	2	15	UUG	0.35	2	UAU	0.25	1

## Data Availability

No data were used to support this study.
